# Humor Styles and Quality of Life in Older Adults Evidence from an Extended Sample

**DOI:** 10.1192/j.eurpsy.2026.11241

**Published:** 2026-07-31

**Authors:** E. Bartzou, C. Tsironis, A. Nakou, E. Dragioti, N. Zagorianakou, S. Mantzoukas, M. Gouva

**Affiliations:** 1 Department of Nursing, School of Health Sciences, Research Laboratory Psychology of Patients, Families & Health Professionals; 2 Department of Nursing, School of Health Sciences, Research Laboratory of Integrated Health, Care and Well-being, University of Ioannina, Ioannina, Greece

## Abstract

**Introduction:**

Humor is an important psychological resource associated with resilience, well-being, and social functioning. In later life, humor may influence quality of life, serving as a coping mechanism against the challenges of aging. Previous research has indicated that positive humor styles are linked with better psychosomatic health in older adults.

**Objectives:**

The present study aimed to expand previous findings by examining the associations between humor styles and quality of life in a larger sample of older adults.

**Methods:**

A total of 608 older adults (aged 65–99 years) participated in this cross-sectional study.

Participants completed demographic information, the Greek version of the Humor Styles Questionnaire (HSQ) and the SF-36 (Short Form Health Survey) to assess quality of life. Descriptive statistics and Pearson correlations were performed.

**Results:**

Self-Enhancing Humor showed the strongest positive correlations with General Health (GH, r=0.38), Social Functioning (SF, r=0.38), Mental Health (r=0.37), Physical Health (r=0.36), and Mental Well-Being (MH, r=0.35). Affiliative Humor was also positively, though more modestly, associated with Social Functioning and Mental Well-Being (r≈0.21). In contrast, Aggressive and Self-Defeating Humor did not demonstrate significant positive relationships with quality of life indicators.

**Conclusions:**

These findings, based on an extended sample, confirm and strengthen previous evidence that positive forms of humor—particularly Self-Enhancing Humor—are closely associated with better physical, mental, and social aspects of quality of life in older adults. Promoting such humor styles may be a valuable target for psychosocial interventions aiming to improve well-being in later life.

**Disclosure of Interest:**

None Declared

